# Supplementary material for: The interactome of intact mitochondria by cross-linking mass spectrometry provides evidence for coexisting respiratory supercomplexes
Source: Mol Cell Proteomics. 2017 Dec 8;17(2):216–32. doi: 10.1074/mcp.RA117.000470 (PMC5795388; doi:10.1074/mcp.RA117.000470)
Supplement: Supplemental Data [file supp_RA117.000470_133922_0_supp_23978_fzffwf.zip › spectra_annotation/mito_spectra_annotation/465-1-6-1-14-1.pdf]

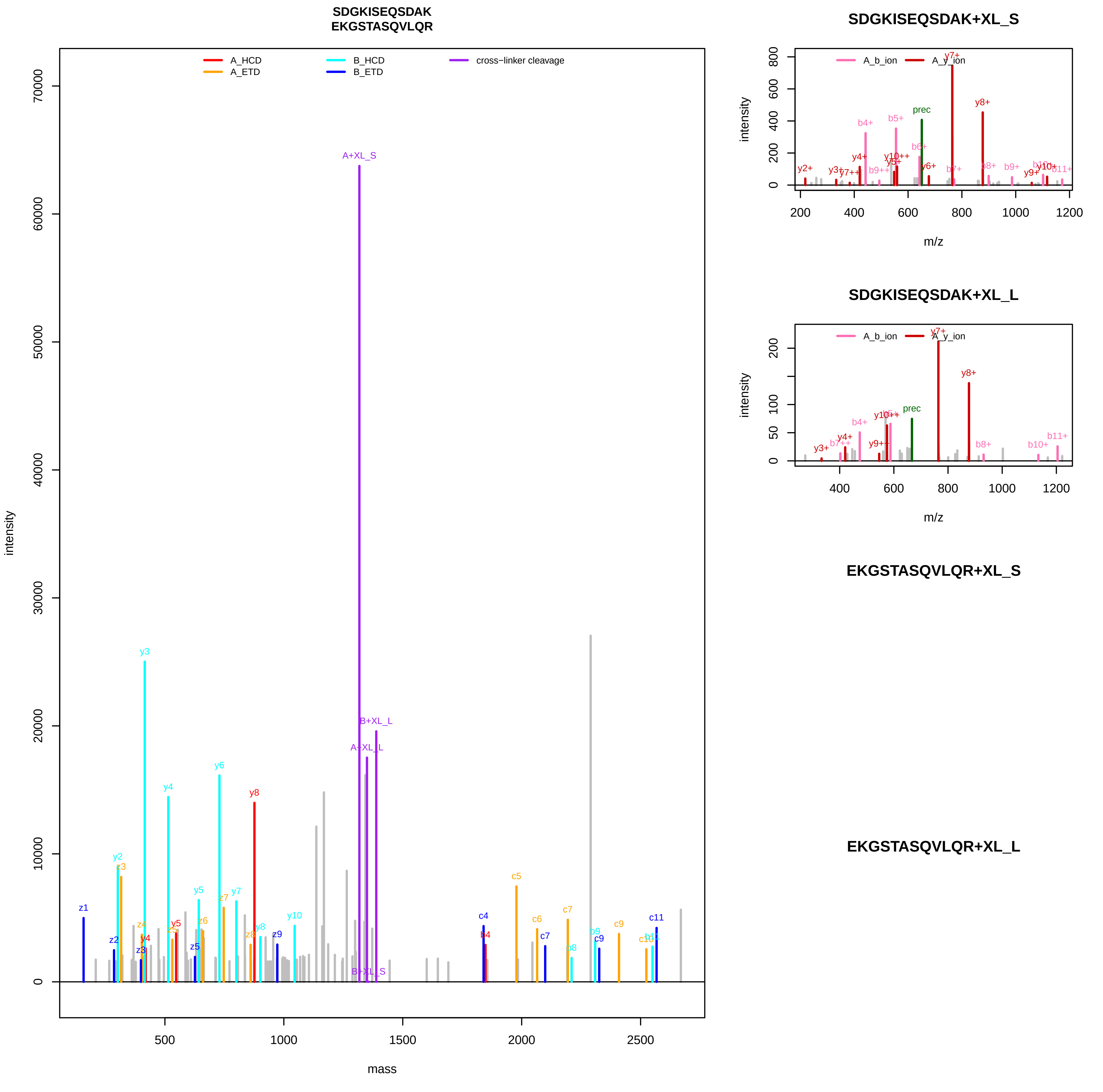

SDGKISEQSDAK
EKGSTASQVLQR
A_HCD
A_ETD
B_HCD
B_ETD
cross−linker cleavage
A+XL_S
B+XL_L
A+XL_L
B+XL_S
y2 z3 y3 y4 y5 y6 y7 y8 y10 z1 z2 z3 z4 z5 z6 z7 z8 z9
c4 b4 c5 c6 c7 b8 b9 c9 c10 b11 c11
intensity
mass
SDGKISEQSDAK+XL_S
A_b_ion
A_y_ion
prec
y2+ y3+ y4+ y7++ b4+ b5+ b9++ y10++ y5+ b6+ y6+ y7+ b7+ y8+ b8+ b9+ y9+ b10+ y10+ b11+
m/z
SDGKISEQSDAK+XL_L
y3+ y4+ b7++ b4+ y9++ y10++ b5+ prec y7+ y8+ b8+ b10+ b11+
EKGSTASQVLQR+XL_S
EKGSTASQVLQR+XL_L
